# Roadmap to tackle antibiotic resistance in the environment under the One Health framework

**DOI:** 10.1002/mlf2.12078

**Published:** 2023-09-03

**Authors:** Liguan Li, Tong Zhang

**Affiliations:** ^1^ Environmental Microbiome Engineering and Biotechnology Laboratory, Center for Environmental Engineering Research, Department of Civil Engineering The University of Hong Kong Hong Kong China

**Keywords:** antibiotic resistance, environment, One Health, roadmap

## Abstract

Antibiotic resistance has been recognized as a major challenge worldwide for humans. “One Health” has been recognized as a key concept for containment of antibiotic resistance. Under the framework, the role of the environment in the development of antibiotic resistance genes (ARGs) has become increasingly obvious. Despite numerous efforts, response to antibiotic resistance is considered to be inadequate, which is probably due to the lack of a clear roadmap. Here, we propose a “One Health” roadmap to combat antibiotic resistance in the environment through (1) understanding environmental resistome. The environmental gene pool has long been recognized as the single largest reservoir of both known and novel ARGs. (2) Standardizing ARG quantification. Systematic joint efforts based on standardized quantification are urgently needed to understand the true tempospatial profiles of the environmental resistome. (3) Identifying mechanisms of resistome development. Horizontal gene transfer and co‐selection have been recognized as the two main mechanisms contributing to the environmental resistome. (4) Establishing a risk‐assessment framework. The first critical step for large‐scale cost‐effective targeted ARG management in the environment is the risk assessment to identify the priority ARGs for control. (5) Formulating regulatory standards. By correlating the environmental ARG profile with public health, we may identify the indicator ARGs that can be integrated into current environmental quality standards. (6) Developing control strategies. Systematic analysis of available control technologies is required to identify the most feasible ones to curtail the spread of ARGs in the environment. The proposed roadmap under the “One Health” framework provides a guide to tackle antibiotic resistance in the environment.

## INTRODUCTION

Widespread antibiotic resistance, together with climate change, environmental damage, and water stress, have been recognized as major challenges worldwide for humans in this century^1^. Yet, effective control strategies of antibiotic resistance are in their infancy as the emergence and dynamic of antibiotic resistance genes (ARGs) remain largely unknown. While the therapeutic use of antibiotics directly affects resistance development, it is increasingly obvious that the environmental dimension of antibiotic resistance must also be addressed. The “One Health” concept has been distinguished as a key element in regional, national, and international strategies for containment of antibiotic resistance^2^. In fact, a peek into the evolution of public health systems in history shows that the concept emphasizing the interdependence between the health of humans, animals, and the environment has long existed, which saved millions of lives and significantly extended the human lifespan. For example, the outbreak of cholera in England in the mid‐19th century and later development of water and wastewater treatment plants are simply due to the fact that contamination of water was closely linked to the prevalence of infectious diseases among the public. By addressing the problem upstream, the introduction of wastewater treatment systems in the era saved huge resources downstream—healthcare costs. Such a philosophy of “upstream thinking” was illustrated by an ancient Chinese story^3^ as well.

*Bian Que was a well‐known physician in ancient China over 2300 years ago. One day, the King asked him “You have two brothers. They are also doctors. Who is the best among you?” Bian Que answered, “My eldest brother is the best, the next is my second eldest brother, and the last is me.” The King was surprised and asked him why. Bian Que replied “My eldest brother treats a disease when people do not even recognize it. That's why he doesn't enjoy wide recognition. My second eldest brother treats a disease when symptoms are subtle, so he is unknown beyond our village. As for myself, I cure people when they are already very sick. People see me manage to save their lives, and think I am the best doctor.”*

Lessons from history are obvious—“upstream thinking” helps prevent great loss. When confronting the hidden pandemic of antibiotic resistance today, we have to respond in the upstream (the environment sector) rather than reacting to it in the downstream (human and animal sectors). The Global Research on Antimicrobial Resistance (GRAM) report estimated that antibiotic resistance was linked to an estimated 4.95 million deaths in 2019^4^. Mitigating antibiotic resistance will have a huge impact on achievement of six of the 17 Sustainable Development Goals (SDGs)^5^. A total of 86 countries have developed and implemented collaborative, multisectoral national action plans in line with the global action to address antibiotic resistance, comprising 44% of the 194 countries in the world^6^. Despite the availability of the action plans, the response to antibiotic resistance is thought to be inadequate. This is probably due to the lack of a clear roadmap, that is, the importance of or the inclusion of “One Health” was not distinctly apparent in the action plans, especially lack of awareness of the environmental dimension of antibiotic resistance. Here, we propose a “One Health” roadmap to combat antibiotic resistance in the environment by understanding the current status, standardizing ARG quantification, identifying mechanisms of resistome development, establishing frameworks of risk assessment, formulating regulatory standards, and developing control strategies.

## UNDERSTANDING THE ENVIRONMENTAL RESISTOME

The environmental gene pool far exceeds that of human and animal microbiota, mainly due to a diversity of ecological niches created by the complex interaction between microorganisms and the environment^7^. The numerous genes in the environment may include candidates that can be acquired by bacteria, especially pathogens, to resist antibiotics. The environment has long been recognized as the single largest source and reservoir of ARGs with both known and novel resistance mechanisms for all approved and probably future antibiotic candidates^8^, ^9^. The role of the environment in ARG emergence and transmission is obvious. With the large number and diversity of bacteria as well as complex microbial and genetic interactions, the environment indeed provides ideal conditions for gene development and exchange between indigenous microorganisms and those from humans and animals. Ubiquitous selective and coselective pressures (e.g., antibiotics, nonantibiotic biocides, and metals) in the environment may further promote ARG transmission and maintenance^10^, ^11^. This complexity, however, is hardly addressed by traditional culture‐based studies, which still represent the current gold standard in clinical microbiology. A large number of studies have explored antibiotic resistance in various environments, mainly ARG profiling by genomic approaches, which provides a more comprehensive picture of antibiotic resistance potential than clinical isolates. Through benchmarking and synthesis of 1427 metagenomic data sets covering 85 countries, including 13 habitats of human and animal feces, wastewater, sediment, and marine water, the huge diversity of ARGs harbored by the environment and significant anthropogenic interference on environmental resistome were uncovered^12^. For example, wastewater‐influent samples have comparable ARG levels as human feces (~2 copies per cell), whereas natural marine water has much lower levels (~0.02 copies per cell). Natural environments have an unexpectedly higher proportion of ARG types, which, however, are rare in the anthropogenic environment, including ARGs against fosfomycin and trimethoprim. It is believed that, compared with our current knowledge, the environment contains far more novel ARGs that have not been sequenced as yet. It is therefore quite possible that the vast uncharacterized microbiota is the origin of those ARGs for which sources have not been identified so far. Through ubiquitous human activities and mobile genetic elements, known and novel ARGs in different ecologies tend to be interconnected and ultimately impact human health^13^.

## STANDARDIZING ARG QUANTIFICATION

Despite numerous individual local‐scale studies, systematic joint efforts using standardized quantification methods are most needed to understand the true temporal and spatial profiles of the environmental resistome. To achieve the goal, the first efforts should be directed toward method standardization for both sampling and analysis. Environmental samples with various features require different sampling and preservation methods for both culture‐dependent and ‐independent analyses. For analysis of antibiotic resistance in the environment, current methods include isolation, polymerase chain reaction (PCR), and metagenomics. Ideally, the three methods complement each other, which will help connect different studies on antibiotic resistance, especially ones linking the environment to the clinic, since isolation is still the common practice in studies on clinical resistance. As the majority of bacteria and genes are unknown in the environment, metagenomics is often the preferred method for environmental studies of antibiotic resistance, which is also the choice for the Global Sewage Surveillance Project^14^. Although the currently popular short‐read metagenome is known to be notoriously uncertain in terms of capturing the genetic context especially from complex environmental samples, the rapid development of long‐read sequencing^15^ and genomic‐crosslinking^16^ may help recover the host and mobility of ARGs. Compared with methods based on traditional isolation and PCR, metagenomic analysis, though popular nowadays, is far less standardized, thus making data comparison and synthesis difficult. We therefore call for the standardization of metagenomic analysis of ARGs in the environment, and suggest starting by focusing on three aspects: (1) the universal quantification unit, which makes direct interstudy comparison possible. ARG copy per cell has been suggested as a universal unit for ARG quantification, as it has more straightforward biological/clinical meaning than other units like RPKM^17^. (2) Absolute quantification, which is fundamental in allowing for comparative evaluation and enabling quantitative risk assessment. The above unit of ARG copy per cell can be converted into absolute quantification by considering total cell number, which can be quantified by flow cytometry, microscopy, or spike‐in methods^18^. (3) Environmental reference sample, which helps evaluate variations caused by factors like DNA extraction, sequencing platforms and operation biases, and thus identifies the true biological variations. More standardization of metagenomic‐based ARG analysis in the future can pave the way for more insightful studies on risk assessment, source–sink relationships, and spatiotemporal trends. This can help understand where and when selection for ARGs primarily occurs, thus aiding in use of appropriate measures to reduce the risk to human health.

## IDENTIFYING MECHANISMS OF RESISTOME DEVELOPMENT

Quantifying fates, deciphering behaviors, and identifying drivers and barriers for ARG transmission in the environment are key elements to understand and manage the antibiotic resistance crisis. In the environment, direct selection is usually very low, almost absent^19^. In many cases, different levels of ARGs in the environment can be simply attributed to fecal pollution, rather than on‐site selection by the antibiotic residues^20^. Moreover, it is widely accepted that critical evolutionary events are rare. Horizontal gene transfer (HGT) and co‐selection have been recognized as the two main mechanisms contributing to propagation of the environmental resistome^21^. Successful ARG acquisition by pathogens is the result of a step‐wise evolution process^22^. The process is not easy; however, some environments like wastewater are probably more likely than others to promote ARG transfer, either through the enrichment of mobile elements or possibly due to the presence of various stresses favoring gene exchanges. Compared to the macro‐environment, microbial interaction and selection pressure in the micro‐environment like biofilm and particle surface are more likely to facilitate ARG dissemination, which, however, has received far less attention. Recent studies have revealed that biocides like certain antibiotics and other pharmaceuticals can accelerate HGT significantly^23^. Under complex microbial interaction and environmental conditions, changes in the genetic context around ARGs can occur frequently, which then may increase transmission potential and the levels of antibiotic resistance. Moreover, metals and nonantibiotic biocides can co‐select for antibiotic resistance through mechanisms of co‐resistance (genes located within proximity) and cross‐resistance (the same gene with different resistance functions)^23^. The co‐selection pressure may further maintain co‐resistance developed among bacteria and thus play a role in ARG persistence in the environment^24^. However, it is unclear to what level HGT and co‐selection in the environment each contributes to the propagation of resistance in clinical pathogens during the current antibiotic era, and more studies are required in the future to examine this.

## ESTABLISHING FRAMEWORK OF RISK ASSESSMENT

Current global prevalence of numerous ARGs calls for scientifically proven targeted mitigation strategies. To achieve the best efficiency, such strategies ideally consider the factors contributing to each step in ARG development including emergence of novel ARGs, transmission pathway, and acquisition by pathogens. However, identification of the target ARGs for control is often challenging as it is not straightforward to distinguish the ARGs of high health concern from those performing common biological functions such as the efflux system and stress signaling^25^, ^26^. Even for those “true” ARGs, their risk to human health depends on factors like the pathogenicity of host bacteria, genetic context, and likelihood of transfer to human pathogens. In fact, only a few ARGs deposited in current databases hosted by human pathogens and with high mobility pose a threat to public health^27^. Thus, the first critical step for the large‐scale cost‐effective ARG management in the environment is risk assessment to identify the priority ARGs for control. A framework based on evaluation of factors of human‐associated enrichment, gene mobility, and host pathogenicity has been recently proposed^27^. The framework identified 73 high‐risk ARG groups, of which 35 were included in the WHO list of high clinical concern^27^. For the risk assessment at specific sites like beach water, quantitative microbial risk assessment using viability‐resolved metagenomic methods integrating risk evaluation of pathogens and ARGs may help us conduct a holistic microbial risk assessment and guide risk management to control antibiotic resistance^28^.

## FORMULATING REGULATORY STANDARDS

With the developed risk‐ranking framework, it will be possible to establish the scientific criteria that do not cause adverse health impacts for those ARGs with high priority. Ideally, by correlating the environmental occurrence of high‐risk ARGs with high‐quality clinical data, we may identify the indicator ARGs used as a proxy for detecting the overall resistance level and health risk of the environment, like *Escherichia coli* and other indicator organisms commonly used in water quality standards. Still, translation from basic environmental ARG studies into public health actions is very challenging, as it requires much more evidence than strong statistical correlations. A possible way is to integrate ARG analysis into the current microbial quality standards. For example, in addition to the basic microbial guideline on *E. coli* concentration, we suggest setting up regulations on the subgroup of *E. coli*'s resistance to a few selected antibiotics for monitoring water quality and managing the discharges from the hotspots like hospital wastewater and animal farm waste. Currently, the technical details to formulate regulatory standards for routine monitoring and auditing are still not clear. This requires long‐term close collaborative efforts between researchers from the fields of public health, environmental science, and engineering.

## DEVELOPING CONTROL STRATEGIES

Mitigating health risks of antibiotic resistance in the environment calls for actions to reduce emission and exposure. Control strategies should be carried out on a broad scale, ideally worldwide, since antibiotic resistance can easily achieve cross‐border transmission in this era of globalization. Given the huge resource demand, these strategies are expected to encounter huge challenges. Priority should therefore be assigned first to identify where mitigation is urgently needed and actions are feasible. Ideally, based on the above risk assessment and the regulatory standard, we can identify those critical hotspots for control. Currently, even without well‐established risk assessment mechanisms and regulatory standards, it is widely accepted that control technologies should be developed to minimize ARG emission from important sources like wastewater treatment plants and animal farms. Installing the basic treatment of municipal/agricultural/industrial wastewater, especially in low‐ and middle‐income countries, can be a starting point as it can significantly reduce emissions of ARGs, also many other biological and chemical pollutants, thus substantially improving water quality^29^. Complementing traditional wastewater treatment with more advanced methods (such as membrane, ozonation, and chlorination) is another step to remove a wide range of contaminants^30^. Due to concerns of disinfection byproducts, the use of chemical methods might be curtailed. Physical methods like membrane units are effective barriers for not only antibiotic resistance bacteria and genes but also other contaminants. Moreover, by replacing biological treatment with membrane separation, we can considerably prevent ARG spread through microbial interaction, which is indeed a major concern for biological wastewater treatment, especially in pharmaceutical industries. Therefore, replacing or enhancing wastewater treatment by rapidly developed membrane technologies yields a “collateral benefit.” Systematic scientific and technical analyses of different control technologies are required to identify the most feasible and efficient methods for specific application scenarios to curtail the spread of antibiotic resistance in the environment.

The environmental resistome reflects clinical antibiotic resistance prevalence, possibly more than has generally been recognized. The proposed roadmap under the “One Health” framework provides a guide to get started on tackling antibiotic resistance in the environment. As the majority of current studies are still at the beginning of the roadmap, that is, understanding the environmental resistome, more actions for the next critical steps including method standardization, mechanistic studies, risk assessment, priority identification, and control measurement should be initiated. Together with efforts in human and animal sectors, we may substantially mitigate and prevent antibiotic resistance in the future and contribute to sustainable development.
